# Hypertension Diagnosis, Treatment, and Control among Older Chinese: Trends in the Hypertension Care Cascade

**DOI:** 10.1093/geroni/igab046.2328

**Published:** 2021-12-17

**Authors:** Erfei Zhao, Qiao Wu, Eileen Crimmins, Yuan Zhang

**Affiliations:** 1 University of Southern California, Leonard Davis School of Gerontology, Los Angeles, California, United States; 2 University of Southern California, Los Angeles, California, United States; 3 University of North Carolina, University of North Carolina, North Carolina, United States

## Abstract

Hypertension is a major risk factor for cardiovascular disease, which is the leading cause of death in China. Older persons are at higher risk of elevated blood pressure and are more likely to have insufficient hypertension care, including delayed diagnosis and poor management. However, we know little about hypertension care among older Chinese at a population level. We use a nationally representative sample of older adults from the China Health and Retirement Longitudinal Study (CHARLS) in 2011 and 2015 (n = 9,083), to clarify the hypertension care cascade for the older population in China by specifying the level of diagnosis, treatment, and control of hypertension. We then examine the characteristics of those (1) who received appropriate hypertension care and (2) whose care improved over time. Diagnosis and care improved between 2011 and 2015. Among those with hypertension, 55% and 67% were diagnosed in 2011 and 2015 respectively; 46% and 60% were treated with modern medication; and 20% and 29% were effectively controlled. Those who had higher income (OR=1.52; P<0.01) or obese (OR=2.43; P<0.001) were relatively more likely to be diagnosed, while those living in the western region (OR=0.65; P<0.01) or living in urban areas with a rural hukou (OR=0.54; P<0.01) were less likely. Persons age 75+ (OR=0.55; P<0.05) were less likely to have their blood pressure controlled, while those who had higher income (OR=1.50; P<0.05) were more likely. The improvement from 2011 to 2015 in hypertension care was concentrated among those that are obese or living in the West.

